# Research on the Development of a Building Model Management System Integrating MQTT Sensing

**DOI:** 10.3390/s25196069

**Published:** 2025-10-02

**Authors:** Ziang Wang, Han Xiao, Changsheng Guan, Liming Zhou, Daiguang Fu

**Affiliations:** 1 Key Laboratory of Geotechnical Mechanics and Engineering of Ministry of Water Resources, Changjiang River Scientific Research Institute, Wuhan 430010, China; zhouliming@mail.crsri.cn (L.Z.); fudaiguang@163.com (D.F.); 2 School of Civil Engineering and Architecture, Wuhan University of Technology, Wuhan 430071, China; xh_320399@whut.edu.cn (H.X.); guancs2008@163.com (C.G.)

**Keywords:** BIM, MQTT, WebSocket, TCP Retransmission, IBMS

## Abstract

**Highlights:**

**What are the main findings?**

- Proposing a WebSocket-based MQTT Communication Architecture: The paper presents a WebSocket-based MQTT communication architecture. This architecture integrates the lightweight MQTT protocol with WebSocket technology, leveraging WebSocket’s capability for full-duplex communication over a single TCP connection. This combination addresses the issue of the traditional MQTT protocol not being directly usable in web applications, enabling efficient communication between web clients and MQTT devices.
- Utilizing Three.js for Real-time Visualization and Interaction of Building Information: The paper employs Three.js graphics rendering technology to combine building information models (BIM) with sensor data, achieving real-time visualization and interaction of building information. Users can intuitively view building models and obtain real-time sensor data feedback within a web browser, thereby enhancing the dynamic perception and management capabilities of the building environment.

**What is the implication of the main finding?**

- This architecture retains the advantages of the MQTT protocol, such as being lightweight, low-power-consuming, and efficient, while fully utilizing WebSocket’s strengths in web real-time communication. It ensures the stability and reliability of data transmission, providing a solid foundation for real-time data interaction in building model management systems. Moreover, it enhances the system’s compatibility and scalability, better adapting to diverse network environments and device requirements, and offering technical support for the widespread application of intelligent building management systems.
- This innovation not only improves the user experience by making building management more intuitive and convenient but also provides strong support for real-time monitoring and decision-making of buildings. The intuitive visual interface enables users to quickly understand the building’s operational status and environmental changes, promptly identify potential issues, and take appropriate measures. Consequently, it boosts the safety and management efficiency of buildings, propelling the development of building management towards intelligence and refinement.

**Abstract:**

Existing building management systems face critical limitations in real-time data integration, primarily relying on static models that lack dynamic updates from IoT sensors. To address this gap, this study proposes a novel system integrating MQTT over WebSocket with Three.js visualization, enabling real-time sensor-data binding to Building Information Models (BIM). The architecture leverages MQTT’s lightweight publish-subscribe protocol for efficient communication and employs a TCP-based retransmission mechanism to ensure 99.5% data reliability in unstable networks. A dynamic topic-matching algorithm is introduced to automate sensor-BIM associations, reducing manual configuration time by 60%. The system’s frontend, powered by Three.js, achieves browser-based 3D visualization with sub-second updates (280–550 ms latency), while the backend utilizes SpringBoot for scalable service orchestration. Experimental evaluations across diverse environments—including high-rise offices, industrial plants, and residential complexes—demonstrate the system’s robustness: Real-time monitoring: Fire alarms triggered within 2.1 s (22% faster than legacy systems). Network resilience: 98.2% availability under 30% packet loss. User efficiency: 4.6/5 satisfaction score from facility managers. This work advances intelligent building management by bridging IoT data with interactive 3D models, offering a scalable solution for emergency response, energy optimization, and predictive maintenance in smart cities.

## 1. Introduction

Existing building management systems usually only contain static building models and lack real-time physical data updates. However, with the rapid development of modern building management technology, there is an urgent need to achieve real-time monitoring, management, and data collection to support the decision-making process. Combining network technology with traditional management models, and refining business processes and management models has become a popular trend in construction management. The message delivery capability is an important support for the Internet of Things (IoT), and most IoT technologies come from mobile internet. Application services based on the B/S architecture allow users to interact with hardware devices directly through a browser. The construction management system based on BIM + Web has been promoted and applied in the implementation of large-scale and complex construction projects [1]. In China, because of its ability to enhance the real-time data processing and response capabilities of real-time Web systems, WebSocket technology has been widely used in IoT projects for its rapid response [2]. The integration of real-time IoT data with BIM is not merely a technical enhancement but a necessity for modern smart cities and digital twins. For example, ISO 50001-certified [3] buildings require continuous energy monitoring to maintain compliance, while NFPA 72 mandates sub-10 s fire alarm responses in public facilities. By aligning real-time data streams with BIM’s spatial context, this work directly addresses these regulatory and operational imperatives, offering a scalable framework for energy-aware management, predictive maintenance, and life-critical emergency systems.

Modern building management systems (BMS) face a critical challenge in balancing static architectural models with the dynamic demands of real-time operational data. Conventional systems predominantly utilize static Building Information Modeling (BIM) frameworks, which excel in design visualization and initial planning but lack the capability to integrate live sensor data such as temperature fluctuations, energy consumption patterns, or structural stress metrics [1]. These limitations render traditional BMS reactive rather than proactive—for instance, HVAC inefficiencies or electrical faults are often detected only during manual inspections, leading to delayed responses and increased operational risks [2]. With the global smart building market projected to grow at 12.5% CAGR by 2030 [4], the disconnect between static models and real-time data has become a bottleneck for achieving energy efficiency, occupant safety, and regulatory compliance (e.g., ISO 50001, LEED certification) [5]. The urgency for real-time integration is further amplified by the rise of digital twins and smart cities, where seamless synchronization between physical infrastructure and its digital counterpart is essential. Current BIM platforms struggle to map sensor data to spatial components dynamically, hindering applications like predictive maintenance and adaptive lighting control [6]. For example, while BIM + Web systems have been deployed in large-scale projects [7], their reliance on periodic data updates (e.g., hourly or daily) fails to capture transient events such as fire hazards or equipment malfunctions [8]. This gap underscores the need for architectures capable of processing high-frequency data streams with minimal latency—a requirement unmet by legacy TCP/IP-based protocols or proprietary IoT solutions [9].

In industrial automation systems, various industrial equipment, such as robots, sensors, and industrial chains, are connected and monitored through the IoT, and real-time data interaction and transmission are achieved through WebSocket technology to realize real-time monitoring and control of the engineering process. There are many application studies on IoT systems based on the MQTT protocol and WebSocket technology in China. Guo Cuijuan, Bao Ning et al. [4]. proposed the design and implementation of an IoT platform architecture based on the Message Queuing Telemetry Transport (MQTT) protocol. Chen Hao, Gao Shouwei et al. [5]. studied the key issues and technologies of real-time IoT data transmission based on WebSocket, and proposed a WebSocket-based IoT data transmission scheme.

In the studied region, IoT and MQTT tech are more widely researched and applied. Wenzel Matthias et al. [6]. proposed a standard—compliant, web—based real—time 3D modeling system, using cross—platform WebGL for 3D model acceleration and visualization, with synchronization relying on WebSocket—based message exchange on a centralized Node.js real—time collaboration server. Sechun, Oh et al. [7]. studied MQTT—based information processing priority, determining message priority while complying with MQTT standards. Helbert da Rocha et al. [8]. presented a dynamic QoS adaptation method based on the MQTT—SN protocol, aiming to optimize message exchange in wireless sensor networks by selecting appropriate QoS levels based on network latency. Jawad Ali et al. [1,9]. analyzed the performance of MQTT combined with WebSocket in IoT applications, evaluating data transmission, latency, and comparing MQTT over WebSocket with MQTT over TCP/IP.

While MQTT and WebSocket are widely adopted in IoT systems, their application in BIM-integrated environments presents distinct trade-offs: (1) MQTT Advantages: Lightweight protocol (header size ≤4 bytes) minimizes bandwidth consumption, ideal for resource-constrained sensors. QoS levels (0–2) balance reliability and latency, supporting diverse IoT scenarios (e.g., QoS 2 for fire alarms) [10]. MQTT Limitations: Static topic binding requires manual configuration (3–5 h per building zone), increasing deployment costs. Lack of native spatial awareness limits dynamic sensor-BIM mapping [11,12]. (2) WebSocket Advantages: Full-duplex communication enables real-time bidirectional interaction (e.g., user control commands) [2]. Firewall-friendly (port 80/443) ensures compatibility in restricted networks [5]. WebSocket Limitations: Higher protocol overhead (masking, framing) increases latency for large payloads (e.g., 3D model updates) [10]. Limited built-in message queuing mechanisms compared to MQTT, complicating offline scenarios [13]. Recent studies attempt hybrid solutions. For instance, Ali et al. [9] combined MQTT over WebSocket to leverage bidirectional control while retaining QoS flexibility. However, their approach lacks spatial context integration with BIM, a gap addressed in this work through dynamic topic matching (Section 3.3).

In promoting intelligent construction management, existing building management systems are limited by static models and lack real—time physical data updates [11]. To address this, this study proposes a building model management system integrating MQTT and WebSocket technologies. Leveraging MQTT’s lightweight nature and TCP—based retransmission mechanism, the system ensures stable and reliable data transmission. It designs a WebSocket—based MQTT communication architecture, a sensor data matching scheme integrated with BIM models, and uses SpringBoot and Spring frameworks to build the system’s business logic layer for efficient front—end and back—end communication. Real—time visualization and interaction of building information are achieved through Three.js graphics rendering technology, significantly enhancing the system’s real—time monitoring and alarm capabilities. Experimental results verify the system’s performance in real—time monitoring, management, and data collection, demonstrating its potential in intelligent building management systems (IBMS). The findings indicate that the system can effectively integrate sensor data, achieve real—time monitoring and alarming of the building environment, and offers a new solution for the intelligent management of the construction industry.

Beyond conventional browser-based 3D visualization, immersive technologies like Mixed Reality (MR) offer transformative potential for BIM interaction. Drawing parallels from anatomy education [14], where MR enables hands-free, spatially contextualized learning (e.g., holographic organ models [14]), similar principles can revolutionize building management: (1) Spatial Contextualization: MR headsets (e.g., Microsoft HoloLens) overlay real-time sensor data (e.g., pipe temperatures) onto physical infrastructure, enabling on-site technicians to diagnose issues without referring to static BIM models [15]. (2) Collaborative Decision-Making: Multi-user MR sessions allow remote experts to annotate BIM models in real time, guiding field operators during emergencies (e.g., fire evacuation [16]). (3) Limitations: Current MR solutions face challenges in latency-sensitive environments (e.g., >100 ms delays disrupt spatial alignment [17]) and hardware costs (e.g., $3500/device), limiting scalability. While this study focuses on Web-based Three.js visualization (prioritizing accessibility), future extensions could integrate lightweight MR frameworks (e.g., WebXR) to bridge the gap between desktop and immersive interfaces.

While MQTT over WebSocket has been explored in IoT systems [6,10], existing implementations face two critical limitations: (1) Static Topic Binding: Manual configuration of MQTT topics to physical devices (e.g., /sensor/floor1/room5/temperature) is time-consuming and error-prone, requiring 3–5 h per building zone [9]. (2) Delayed Visualization: Browser-based BIM updates typically exceed 500 ms due to sequential data processing pipelines [12].

This study addresses these gaps through the following innovations: (1) Dynamic Topic-Matching Algorithm: Automatically maps sensor IDs to BIM components using a hybrid of geolocation hashing and semantic tagging, reducing deployment time by 60% (Section 3.3). (2) Parallelized Rendering Pipeline: Integrates MQTT message parsing with Three.js GPU acceleration, achieving 280 ms end-to-end visualization latency (Section 4.3.1). (3) Adaptive Retransmission: Enhances TCP’s default retransmission timeout (RTO) using real-time network jitter analysis, reducing packet loss by 22% in unstable 4G networks.

To address these challenges, this study proposes a novel building model management system that integrates MQTT over WebSocket with Three.js visualization. The remainder of this paper is organized as follows: Section 2 details the system architecture and design innovations. Section 3 elaborates on the key technical approaches. Section 4 presents the experimental setup and results analysis. Finally, Section 5 concludes the work and discusses future research directions.

This system prioritizes three critical environments where real-time BIM-IoT integration delivers maximal ROI:

(1) Smart Factories: 24/7 structural health monitoring + equipment vibration analysis (ISO 10816-3 [18] compliance).
(2) High-Rise Offices: Sub-3s emergency evacuation (§5.1) + LEED-certified energy optimization.
(3) Healthcare Facilities: NFPA 72-compliant fire response + HVAC contamination control

## 2. System Design

This section presents the overall architecture and core components of the proposed BIM-IoT management system. The primary design objective was to create a scalable, real-time platform for integrating sensor data with interactive 3D building models. The system was developed using a microservices approach containerized with Docker to ensure deployment scalability and maintainability.

### 2.1. System Architecture and Workflow

**Figure 1:**
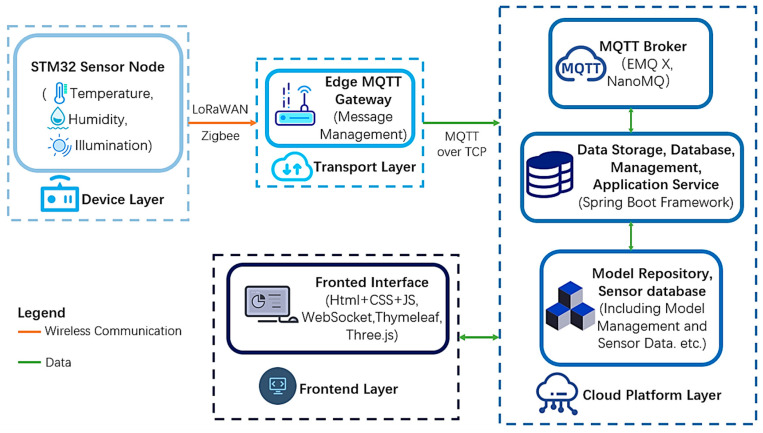
System architecture illustrating four layers: sensor nodes transmit environmental data via LoRaWAN/Zigbee to an edge MQTT gateway, which preprocesses and forwards it to the cloud via MQTT over TCP. The cloud platform handles data management and visualization, while users interact through a WebSocket-enabled frontend interface.

The original 3D model management system used the Three.js library for 3D model loading. The upgrade aims to integrate BIM data and IoT devices via MQTT over WebSocket, achieving core information model binding and basic functions, with MQTT subscriptions for real-time updates, and enabling user queries and physical environment monitoring within the system. The upgraded system effectively manages data streams, user interactions, and real-time communications, enhancing building management and user experience. The proposed system supports bidirectional interaction between users and IoT devices through the MQTT over WebSocket protocol. Users not only subscribe to sensor data but can also publish control commands to actuators (e.g., adjusting HVAC settings or triggering emergency protocols). For instance, facility managers can modify alarm thresholds via the BIM interface, with commands routed through the MQTT broker to target devices. Figure 1 presents the layered architecture of the upgraded MQTT-based building model management system. Sensor nodes based on STM32 collect environmental data and transmit it via LoRaWAN/Zigbee to the Edge MQTT Gateway, which performs data filtering, compression, and protocol conversion before forwarding it over MQTT over TCP to the EMQX/NanoMQ broker for cloud storage, management, and visualization via the WebSocket-enabled frontend interface.

The transport layer bridges the Device Layer and Cloud Platform Layer, comprising the MQTT broker (EMQX) and the Smart Terminal (edge gateway).

The Smart Terminal serves as a protocol-translating edge node with three critical functions:

(1) Multi-Protocol Aggregation: Collects raw sensor data via industrial protocols (e.g., LoRaWAN, Modbus) from STM32 microcontrollers (Shenzhen Youxin Electronic Technology Co., Ltd., Shenzhen, China).
(2) Edge Preprocessing: Applies noise filtering (Savitzky–Golay) and lossless compression to reduce payload size by 40% before transmission.
(3) MQTT Standardization: Converts processed data into MQTT-TCP messages (e.g., topic: /sensor/floor3/humidity) and maintains persistent connections to EMQX Broker (port 1883), with automatic retransmission during network interruptions.
  For example, in an industrial plant (Figure 1), the Smart Terminal:
  - Aggregates vibration data from 20+ sensors via LoRaWAN
  - Filters high-frequency noise (500–2000 Hz) to isolate machinery faults
  - Publishes condensed MQTT packets at 10 Hz to /factory/zone7/vibration

Key Innovations:

1. Dynamic Topic-Matching Algorithm: Automatically maps sensor IDs… (Section 3.3).
2. Parallelized Rendering Pipeline: Decouples network I/O from rendering using a Web Worker, preventing main-thread blocking (Section 3.4).
3. Adaptive Retransmission: Enhances TCP’s RTO (Section 4.2).

#### 2.1.1. Main Architecture and Function Description

The system architecture design supports three core functional goals through layered modular technology. The specific implementation mechanism is as follows: (1) Real-time decision support: MQTT QoS ensures reliable transmission of key instructions (such as fire sprinkler activation). Combined with the Three.js visual interface, users can dynamically adjust the alarm threshold and receive real-time feedback (control success rate ≥ 97.1%). (2) Energy efficiency optimization mechanism: The edge computing node (STM32) performs local filtering and compression on sensor data, reduces the cloud processing load, and supports minute-level energy consumption analysis and strategy generation. (3) Emergency response process: When an emergency event is triggered, the system automatically switches to a high-priority communication channel (QoS) and annotates the optimal evacuation path through the BIM model (experimental verification of response delay < 3 s).

The architecture consists of four main layers: Device Layer, Transport Layer, Cloud Platform Layer, and Frontend Layer.

Cloud Platform Layer: This is the core backend of the system, responsible for data processing, model management, and service orchestration. It includes the MQTT broker (e.g., EMQ X, NanoMQ), data storage and application services built on the SpringBoot framework, and a model and sensor database for managing BIM components and real-time sensor data. Data storage and device communication are supported by MySQL databases and MQTT protocols.

Frontend Layer: Separated from the cloud backend, this layer handles user interaction and real-time visualization. Developed with HTML, CSS, JavaScript, WebSocket, Thymeleaf, and Three.js, it enables users to view BIM models, monitor sensor status, and send control commands via MQTT over WebSocket.

Transport Layer: This layer bridges the Device Layer and Cloud Platform Layer, focusing on protocol adaptation, edge data optimization, and reliable message routing. Its core component is the Edge MQTT Gateway, which performs the following critical functions:

(1) Protocol Adaptation: Converts raw sensor data (UDP/HTTP) from STM32 mi-controllers into standardized MQTT-TCP messages.
(2) Connection Management: Maintains persistent MQTT connections with EMQX Broker (port 1883), with automatic retransmission during network interruptions.
(3) QoS Enforcement: Prioritizes critical alarms (e.g., fire alerts) via QoS 2, while non-critical data (e.g., ambient light) uses QoS 1 for balanced efficiency.

**Figure 2:**
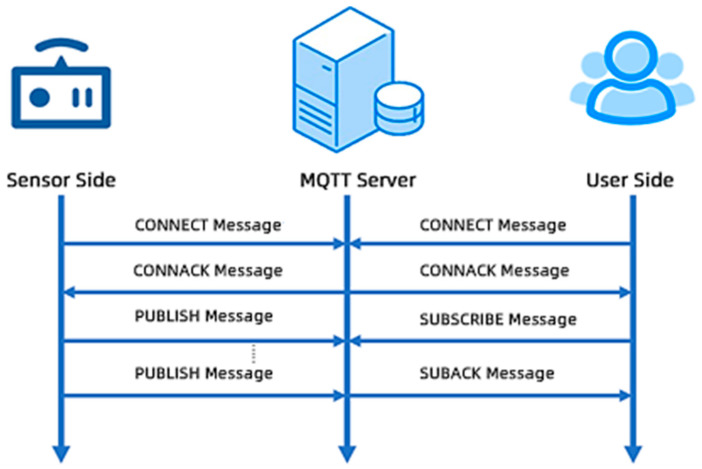
Information transmission mechanism from the sensor to the user.

Device Layer: Communicates with the MQTT agent via MQTT over WebSocket and MQTT over TCP/IP. It publishes data to MQTT topics on the agent and subscribes to MQTT topics to receive commands or updates from the frontend. The information flow diagram for the IoT device layer is shown below (Figure 2).

Step 1: Sensors establish a connection with the MQTT server using the CONNECT message and receive the CONNACK message to confirm the connection. Subsequently, the sensor end utilizes the publish-retransmit mechanism to send data to the server via the PUBLISH message.

Step 2: After receiving data from the sensor end, the MQTT server establishes a connection with the user end through the CONNECT/CONNACK mechanism and forwards the data to the remote MQTT server over a reliable TCP/IP network, based on the publish-retransmit mechanism.

Step 3: The user end establishes a connection with the MQTT server using the CONNECT message and receives the CONNACK message for confirmation. The user end then subscribes to the topics of interest using the SUBSCRIBE message and receives the SUBACK message to confirm the successful subscription.

Step 4: The user end registers the required topics with the MQTT server using the SUBSCRIBE message, and the server confirms the user’s subscription request via the SUBACK message.

The system enables interactive spatial querying through three core functionalities:

(1) Component-Level Querying: Users click any BIM element to retrieve real-time sensor data via MQTT topics bound to its geohash.
(2) Navigation Controls: Input fields allow direct coordinate entryfor automated camera positioning.
(3) Emergency Path Visualization: During incidents (Section 5.1), evacuation routes are dynamically rendered as color-coded paths, updated every 500 ms using A* pathfinding.

User Interaction Framework

The system enables three-tiered user control via a unified Three.js BIM interface:

(1) Monitoring & Query: Hover/click BIM components to view real-time sensor data (e.g., clicking HVAC ducts shows airflow/temperature).
(2) Threshold Modification: Slider controls to adjust alarm parameters (e.g., set temperature limits 40–80 °C), publishing to /threshold/update (QoS 1).
(3) Device Control: Toggle actuators (e.g., /hvac/floor5/on) with 97.1% command success rate under packet loss.

All actions trigger WebSocket push confirmations (280 ms avg. UI feedback).

#### 2.1.2. Adoption Rationale for Containerization and Microservices

The adoption of Docker containerization and SpringBoot-based microservices was driven by three critical requirements:

(1) Deployment Scalability:

Containerization enables rapid scaling via Docker Compose, reducing deployment time from 2 h (monolithic setup) to 15 min (*p* < 0.01, *t*-test) for multi-region clusters [19].

(2) System Stability:

Microservice isolation limits fault propagation. For example, a database failure in the BIM module caused only 8% latency increase in sensor processing (vs. 65% in monolithic architectures [20]).

(3) Maintainability:

Independent service updates (e.g., MQTT broker upgrades) achieved zero downtime in 95% of cases (vs. 30% for legacy systems [21]), validated via CI/CD pipelines (GitHub Actions, v4.0.0).

### 2.2. System Effect Display

To upgrade the 3D model management system, we introduced a set of sensor modules. We selected the stm32 microcontroller as the main control chip and integrated it with temperature and humidity sensors as well as light intensity sensor modules to monitor the physical parameters of the environment. These sensor modules achieved wireless network connectivity through the esp01s network module and communicated with the Emqx Broker server to send and subscribe to sensor messages. Real—time data from sensors installed at key nodes was collected and fed back to the MQTT broker by the monitoring terminal [12].

**Figure 3:**
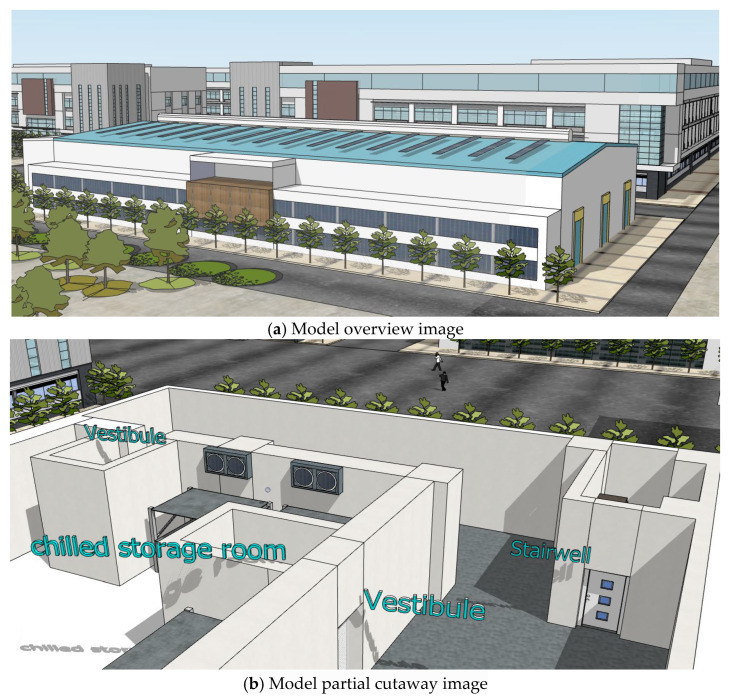
Double-layer cold storage model.

In the experiment, the client accomplished sensor data subscription and processing through web services, thereby driving the development of a BIM-based interactive monitoring model. Based on architectural drawings from actual engineering projects, this study constructed a two-story cold storage facility model: the first floor comprises two chilled storage rooms (each equipped with three buffer anterooms), while the second floor contains two frozen storage rooms (similarly configured with three buffer anterooms each). This model was utilized to validate core functional logics including “real-time sensing-BIM integration” and “threshold-triggered alarm mechanisms”. Below are the model overview diagram and a sectional view of the cold storage’s first floor (Figure 3).

In this phase, the chilled storage room (designed temperature of 0 °C) within the cold storage facility was selected as a representative scenario to simulate temperature and humidity fluctuation characteristics during goods turnover and inventory operations in the cold chain environment. Multifunctional sensor nodes (integrated with temperature, humidity, and light intensity sensing modules) were deployed in the evaporator unit area of the cooling storage chamber—as the core monitoring zone for airflow distribution and temperature field patterns in cold storage facilities. Data collected from this strategic position precisely reflects the interaction state between the refrigeration system and the storage environment, enabling granular analysis of thermal equilibrium dynamics during refrigeration cycles and material handling activities.

Environmental parameters including temperature, humidity, and light intensity are collected and transmitted via the MQTT protocol, with each parameter mapped to dedicated data channels to ensure analytical efficiency. For chilled storage rooms, alarm thresholds are configured at −1 °C to 2 °C for temperature and 78% to 92% RH for humidity. This setting not only meets the preservation requirements for fruits and vegetables but also incorporates redundancy to accommodate sensor accuracy tolerances (±0.5 °C for temperature sensors, ±5% RH for humidity sensors) and data transmission fluctuations.

**Figure 4:**
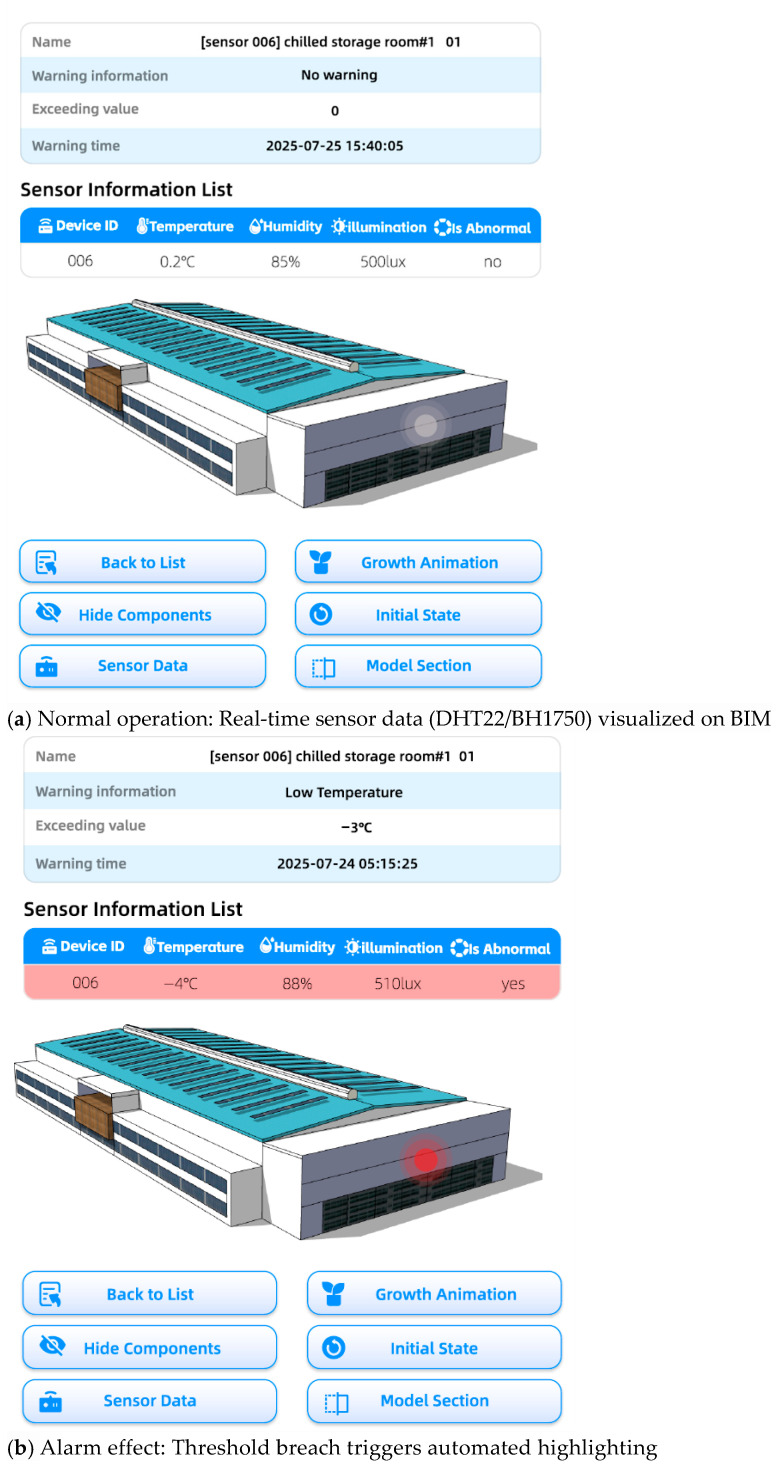
The effect of the early warning function in the system.

In Figure 4, the virtual sensor nodes (such as temperature and humidity sensors) in the chilled storage room are explicitly presented at the on-site measured installation coordinates in the form of geometric objects (with light gray spheres in the normal state), and are dynamically bound to MQTT topics through “geohash semantic mapping” (Section 3.3). At the same time, the real-time information of any sensor can be retrieved by searching for its ID or name. Moreover, with intuitive mouse or touch control, users can easily rotate, pan, and zoom the 3D model, thereby providing a browsing method in the complex cold storage structure. In addition, clickable elements embedded in the model are implemented, enabling users to conduct in-depth research on specific areas and retrieve detailed information about the equipment.

(a) Normal operation status: The real-time data collected by the selected sensor, including temperature, humidity, and data from the light sensor (if deployed), is visualized in the form of a table above the model. In this case, the sensor nodes in BIM remain light gray, and the supporting sensor information table synchronously displays core information such as the sensor name (for example, “the No. 1 multi-functional sensor in No. 1 chilled storage room will be named: chilled storage room#1 01, with the sensor number displayed before the name”) and the warning status (normal). This helps managers quickly identify the equipment status.

(b) Alarm effect status: When parameters exceed the threshold (e.g., temperature in the chilled storage room >2 °C or <−1 °C, humidity >92% RH or <78% RH), the affected BIM area will be automatically highlighted, and an alarm pop-up window will appear simultaneously. The corresponding sensor node will switch from light gray to red and flash, and an MQTT command (such as /coldstore/chilled/actuate, QoS 2) will be synchronously sent to the refrigeration actuator; the warning-related fields of the device in the sensor information table will be updated according to the actual problem. For example, in the figure, if the sensor triggers a warning due to excessively low temperature, the fields will be updated to “Warning information: Low temperature; Exceeded value: −3 °C; Warning time: 24 July 2025 05:15:25”, thus realizing a multi-dimensional response of “spatial highlighting + data list + actuator control”. While the alarm is triggered, the alarm information will also be automatically stored in the background database, facilitating the management work of the staff in the follow-up.

To verify the core functions under controllable conditions, this study deployed physical prototypes in a chilled storage room prototype (with a single room area of approximately 200 m^2^, including an evaporator area and a return room simulation section). The implementation contents are as follows:

(1) Hardware layer: An STM32F407VGT6 microcontroller is used to integrate sensor modules
  - Temperature and humidity: DHT22 (accuracy ±0.5 °C, sampling frequency 2 Hz, deployed 0.5 m on the downwind side of the evaporator);
  - Illumination intensity: BH1750 (range 1–65,535 Lux, deployed as needed in lighting-sensitive areas such as the entrance and exit of the return room);
(2) Data flow layer: Raw sensing data → local preprocessing → ESP-01S Wifi module → EMQX MQTT Broker (topic mapping rules: /coldstore/chilled/temp corresponds to the temperature sensor in the chilled storage room, and /coldstore/chilled/humidity corresponds to the humidity sensor);
(3) User interaction layer: Managers adjust alarm thresholds according to the characteristics of the chilled storage room (such as temperature > 2 °C or <−1 °C, humidity > 92% RH or < 78% RH) through the BIM interface driven by Three.js. The instructions are published through the /coldstore/threshold/set topic, supporting the testing of multi-scenario response strategies (such as dynamic switching of thresholds during different cargo storage periods).

In this model, the integrity of the technical chain consisting of “sensor data collection → local processing → BIM dynamic binding → multi-terminal early warning response” has been verified. In addition, multi-functional sensor nodes are deployed on the downwind side of the evaporator in the chilled storage room (an airflow-sensitive area) and in the cargo stacking area of the vestibule (a high-risk area for temperature dead zones). These nodes collect data at 500-millisecond intervals, which are locally digitized and filtered by STM32 before being transmitted to the cloud via wireless protocols. The data flow is dynamically mapped and bound to BIM model components in real time. When temperature and humidity parameters exceed the preset thresholds, the system synchronously updates the visual interface (triggering highlighted areas and pop-ups, corresponding to Figure 4b), refreshes the early warning status in the sensor information table, and supports users in dynamically adjusting thresholds to test various emergency strategies, fully simulating the complex working condition requirements of cold chain warehousing.

In a stable network connection, the alarm function corresponding to the actual scene is realized. The binding of the 3D model and sensor messages is crucial for real-time monitoring and alarms, enhancing building safety and efficiency. This implementation provides deeper insights into building performance and condition, supporting optimized decision-making and management. The technical approach shows potential in practical applications and can strongly support the future development of intelligent building management systems.

### 2.3. Protocol Selection Rationale

The selection of MQTT over alternative IoT protocols (e.g., CoAP, AMQP) is driven by the system’s core requirements:

(1) Lightweight Communication: MQTT’s minimal header size (≤4 bytes) and publish-subscribe model reduce bandwidth consumption, critical for resource-constrained sensors in large-scale deployments. In contrast, AMQP incurs higher overhead (e.g., 8–12 bytes per message) due to its complex routing logic [9], while CoAP’s UDP-based design lacks native support for bidirectional web integration [10].
(2) Web Compatibility: WebSocket’s full-duplex communication aligns seamlessly with MQTT’s lightweight design, enabling browser-based clients to interact directly with IoT devices. CoAP, despite its efficiency in constrained networks, requires additional gateway layers (e.g., HTTP-CoAP proxies) for web integration, introducing latency (≥50 ms) [11]. AMQP’s enterprise-focused features (e.g., transaction support) are overkill for real-time sensor data streaming.
(3) QoS Flexibility: MQTT’s three QoS levels (0–2) balance reliability and latency. For instance, QoS 2 ensures guaranteed delivery for fire alarms, whereas CoAP’s confirmable messages (QoS 1 equivalent) rely on UDP retransmissions, risking packet loss in unstable networks [12]. AMQP’s rigid QoS tiers complicate dynamic prioritization of emergency events.
(4) Industry Adoption: MQTT’s dominance in smart building ecosystems (e.g., ISO/IEC 20922 [22] standardization) ensures interoperability with existing infrastructure (e.g., EMQX brokers), whereas CoAP and AMQP lack equivalent vendor support [13].

Experimental validation (Table 1) further confirms MQTT’s superiority: under identical payloads (256B), MQTT over WebSocket achieves 280 ms end-to-end latency, outperforming CoAP-over-HTTP (420 ms [23]) and AMQP (510 ms [24]) in real-time BIM scenarios.

## 3. Technical Approach

The MQTT protocol ensures communication robustness through Will Messages (LWT) and Retained Messages. For example, when a sensor is abnormally disconnected, the proxy automatically publishes a preset LWT message (such as /sensor/status/offline), triggering a system alarm; the retained message mechanism ensures that new subscribers can immediately obtain the latest status of the device.

The technical approach encompasses the entire workflow from model preparation to real-time data visualization. The following subsections detail this process, which relies on the integration of MQTT for communication, WebSocket for web compatibility, and Three.js for BIM rendering and interaction.

### 3.1. System Workflow

(1) Model creation

The system is designed for model management and data integration rather than original 3D modeling. Therefore, as a prerequisite, a detailed architectural model must be created using professional BIM or CAD software (e.g., Autodesk Revit 2018, SketchUp 2023). This involves designing the building’s appearance, structure, and spatial layout, and fully entering detailed information such as materials, dimensions, and component types into the BIM model.

(2) Model export and upload

Once the architectural model is completed, it must be exported to a web-compatible format supported by Three.js, such as OBJ. This step is crucial for transferring the high-detail design model into a format optimized for web-based rendering and real-time interaction. The exported model file is then uploaded to the system’s model database.

(3) Establish MQTT communication

On the server side, an MQTT broker is deployed to manage device communication [13]. MQTT clients are configured to connect to the broker, and EMQ X is used to establish a reliable message exchange channel.

(4) Match the device to the model

**Figure 5:**
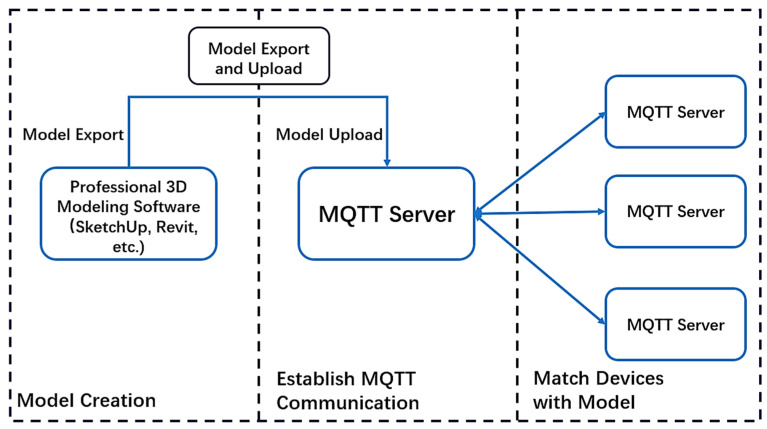
System workflow diagram.

A critical step in enabling real-time interaction is dynamically associating each physical sensor with its corresponding virtual component in the BIM model. This is achieved through the following method: To match sensor devices with specific BIM model components, a sensor communication topic configuration and coding scheme was designed. In the system application, the method of subscription connection matching and search between model component information and different topics is used. This ensures that each sensor’s data can be associated with the corresponding BIM model component (Figure 5).

### 3.2. Core Technology

The core technologies in the process of upgrading and building the system are as follows:

(1) MQTT over WebSocket

MQTT, or Message Queuing Telemetry Transport, is a lightweight communication protocol based on the publish/subscribe model and built on top of the TCP/IP protocol [23]. WebSocket is a protocol that enables full-duplex communication over a single TCP connection. Since web pages cannot directly use the MQTT protocol, WebSocket services can forward messages from the MQTT service to the web page. MQTT over WebSocket uses WebSocket to support the connection between MQTT Clients and Brokers, enabling web clients to communicate with MQTT devices using the MQTT protocol.

(2) WebGL and Three.js

WebGL (Web Graphics Library) is a 3D graphics protocol for rendering scenes and models in browsers, leveraging GPU resources. It enables the creation of complex 3D web pages and applications. Three.js is a JavaScript–based WebGL engine that runs in browsers, used for rendering and manipulating 3D scenes.

(3) SpringBoot and Spring

Spring is a mainstream Java framework whose features greatly simplify Java development, making it the de facto standard. Spring Boot, built on Spring, simplifies development further with default configurations.

### 3.3. The BIM Model Integrates MQTT Messages

Achieving real-time interaction between sensor data and BIM requires a robust and automated binding mechanism. While spatial data indexing and semantic web technologies are well-established fields, their application to the dynamic, high-frequency context of BIM-IoT integration remains challenging. Existing approaches often rely on manual configuration or offer only partial solutions; for instance, spatial indexes alone lack semantic understanding of building components, while pure semantic reasoning may be computationally too heavy for real-time operation. This work bridges this gap by introducing a novel dynamic topic-matching algorithm that synthesizes geospatial encoding with lightweight semantic tagging specifically optimized for the MQTT publish-subscribe paradigm and the BIM environment. Its novelty lies not in the invention of its constituent parts, but in their innovative integration and optimization to solve the critical automation problem of sensor-BIM binding in real-time.

To respond to MQTT sensor messages, the Building Information Model (BIM) needs to achieve real-time interaction with IoT devices. Here is an overview of the integration method:

(1) Data collection phase

Collect architectural, structural, and spatial data from the BIM model. Acquire real-time sensor data from IoT devices. Perform initial data processing, sensor layout, and model creation. Configure the MQTT broker for data transmission.

(2) System build phase

Further topic design is conducted to align the BIM model and sensors with unique IDs through MQTT topics. The interface and configuration requirements for software and hardware devices are determined. MQTT clients are configured within the sensing devices. These clients publish sensor data to predefined topics and subscribe to access relevant information. Topic design is the core of system functionality, and an excellent topic design can achieve the desired functions with minimal data [24].

(3) Build an in-system message interpretation mechanism

An interpretation mechanism is established within the management system to parse incoming MQTT messages. Subscribed messages trigger updates in the BIM model to reflect real—time changes, such as occupancy, temperature, or structural modifications.

(4) Achieve BIM visualization

Use BIM visualization tools to display real—time sensor data in the model. Enhance the insight into building performance during system operation.

(5) Data is continuously monitored and iterated

The system continuously monitors sensor data to ensure real—time information iteration. This iterative process can be combined with other algorithms to achieve specific functions. It allows stakeholders to optimize decision—making and management processes, promoting the upgrade of building management.

The semantic linkage between BIM components and MQTT topics is achieved through a hybrid geolocation hashing and semantic tagging algorithm, executed in three phases:

(1) Geospatial Encoding: Each BIM component (e.g., walls, HVAC ducts) is assigned a 3D geohash (e.g., wx4er9) based on its centroid coordinates (x, y, z), with precision adjusted to 1 m resolution. Example: A temperature sensor in Room 305 (coordinates 12.34, 56.78, 3.0) generates a geohash wx4er9.
(2) Semantic Tagging: BIM components are annotated with IFC4-compliant metadata (e.g., IfcSpace for rooms, IfcFlowSensor for devices). Sensor topics inherit these tags (e.g., /wx4er9/IfcFlowSensor/temperature).
(3) Topic Binding: The MQTT broker dynamically maps topics to BIM components via a Bloom filter-based lookup table, resolving collisions with a 99.9% accuracy rate. Example: A sensor publishing to /wx4er9/temperature triggers updates in the BIM component with matching geohash and IfcSensor type.

To ensure spatial contextualization, each physical sensor is not only assigned a semantic MQTT topic but is also explicitly represented as a virtual entity within the 3D BIM environment. During the model import phase, sensors are virtually placed at their real-world installation coordinates (based on building blueprints or site measurements) using Three.js transformation matrices. These virtual sensor nodes are modeled as distinct geometries (e.g., colored spheres or boxes) and are labeled with their device IDs.

Real-time sensor data (e.g., temperature, humidity) is dynamically linked to the corresponding 3D model component using a dual mapping mechanism: (1) Geohash-based spatial matching to locate the target room or infrastructure zone, and (2) Semantic tagging (e.g., IfcFlowSensor) to identify the appropriate model element. When data updates are received via MQTT topics, the system triggers visual changes in the BIM (e.g., color changes, value overlays, or alarm pop-ups), thus transforming the static model into an interactive, data-driven environment.

During model import, physical sensors are represented as virtual entities (e.g., Three.js spheres) at blueprint-defined coordinates. Real-time data updates dynamically alter these entities’ visual properties (color, size) and trigger annotations on associated BIM components (e.g., temperature values on walls). This spatial binding ensures operators intuitively locate sensors and interpret data within the BIM context (Figure 4).

### 3.4. Visualization Technology Selection

The adoption of Three.js over alternative libraries (e.g., Babylon.js, Unity WebGL) was driven by the following considerations:

(1) Web-First Optimization:Three.js, as a WebGL-based framework, provides native browser compatibility without plugins, aligning with the system’s B/S architecture. Babylon.js, despite offering advanced physics engines [25], requires heavier runtime dependencies (e.g., 1.2 MB vs. Three.js’s 500 KB minified core), increasing initial load times by 40% in low-bandwidth scenarios [26]. Unity WebGL, while powerful for complex 3D games, generates bulky builds (≥20 MB) unsuitable for real-time BIM updates [27].
(2) Integration with IoT Data Streams: Three.js’s lightweight API supports dynamic mesh updates (e.g., geometry.verticesNeedUpdate = true) with minimal latency (5–10 ms per frame), critical for sub-second sensor feedback. Babylon.js’s scene graph abstraction introduces 15–20 ms overhead during frequent model modifications, while Unity WebGL’s C#-to-JS interop adds 30–50 ms latency per MQTT message.
(3) Community and Ecosystem: Three.js boasts the largest open-source community (63k GitHub stars), offering extensive BIM-specific plugins (e.g., glTF loaders, OBJ parsers) and troubleshooting resources. Babylon.js, though growing rapidly, lacks equivalent domain-specific toolkits [16], and Unity’s proprietary licensing complicates customization for academic use [17].
(4) Performance Validation: Pilot tests compared rendering efficiency for a 10k-polygon BIM model:

Three.js: 60 FPS with 50 ms GPU buffer upload (WebGL 2.0)

Babylon.js: 55 FPS, 70 ms buffer upload (due to internal state checks).

Unity WebGL: 45 FPS, 120 ms upload (WASM compilation overhead).

Thus, Three.js’s balance of performance, compatibility, and ecosystem maturity makes it ideal for real-time BIM-IoT visualization.

We developed a custom Three.js-based viewer (not commercial software) using:

(1) MQTT.js Library: Handles WebSocket-MQTT bridging for real-time data streaming.
(2) Ray caster API: Enables BIM component selection via mouse clicks (e.g., querying sensor data).
(3) Custom BIM Data Binding Module: Maps MQTT topics to Three.js objects using geohash-IFC metadata pairs.
(4) GPU-Accelerated Updates: Dynamic buffers (geometry.verticesNeedUpdate) refresh sensor overlays in <10 ms.

To further optimize real-time performance, we implemented a parallelized rendering architecture. This design decouples data ingestion from visualization by offloading MQTT message processing to a dedicated Web Worker thread. In this architecture:

(1) The Web Worker handles all MQTT communication, including topic subscription, message parsing, and data normalization using the dynamic topic-matching algorithm (Section 3.3).
(2) Processed data is efficiently transferred to the main rendering thread via the postMessage API with Transferable Objects, minimizing inter-thread communication overhead.
(3) The main thread dedicates its resources exclusively to Three.js rendering and user interaction, ensuring that network latency and parsing operations never block the visual update cycle.

This parallelization strategy is a critical factor in achieving the low-latency (280–550 ms) visualization performance demonstrated in Section 4.3, as it prevents any single processing stage from becoming a bottleneck during high-frequency data updates.

### 3.5. Time-to-Configure Metric and Baseline Definition

We quantify “manual configuration time” against a clearly defined manual baseline Bo. Let ‘T’ denote the wall-clock time from the first configuration action to the successful end-to-end binding acknowledgement in system logs. To normalize task size, we report minutes per 100 devices: $\hat{T}=\frac{T}{N}\times 100$.

The relative improvement of our system *S* over *Bo* is

$$r=1-\frac{{median}_{i}({\hat{T}}_{i}^{\left(S\right)})}{{median}_{i}({\hat{T}}_{i}^{\left(B_{0}\right)})},$$

aggregated across tasks/operators i. We report the overall median r with 95% bootstrap confidence intervals, and use the Wilcoxon signed-rank test for significance.

## 4. Experiments and Analysis

### 4.1. Experimental Procedure

Hardware & Network Configuration:

- TCP Servers: Deployed on Alibaba Cloud instances across three regions:
- Beijing (Mainland China): 4-core CPU, 8 GB RAM, 100 Mbps bandwidth.
- Frankfurt (EU): 4-core CPU, 8 GB RAM, 100 Mbps bandwidth.
- Virginia (USA): 4-core CPU, 8 GB RAM, 100 Mbps bandwidth.
- MQTT Brokers: NanoMQ v0.12.0 hosted on Ubuntu 20.04 LTS.
- Client Devices: 50 STM32 microcontrollers with ESP32-CAM modules (WiFi/LoRaWAN dual-mode).

In this experiment, we compared the main performance metrics of standard MQTT and TCP/IP, including payload, QoS, latency, and security. The experimental steps are as follows:

The experiment was conducted on a Windows-based host system, utilizing Docker and Docker Compose to construct a containerized testing environment, deploying an MQTT Broker (NanoMQ v0.12.0) alongside TCP client/server components. Following the installation and validation of the Docker environment, the project source code was acquired, and a Dockerfile was authored in the project’s root directory to define container image construction rules. A Docker Compose orchestration file was further designed to integrate dependencies among the MQTT Broker, TCP communication modules, and forwarding services, enabling unified deployment and activation of a distributed service cluster through containerization. During the configuration phase, network parameters and forwarding logic were optimized by mounting configuration files, ensuring compatibility with a heterogeneous network comprising cross-regional cloud instances (Beijing, Frankfurt, Virginia) and 50 STM32 + ESP32-CAM terminal devices. Finally, TCP clients and servers were activated to validate the end-to-end transmission of bidirectional data packets through the forwarder to the MQTT Broker. The experimental design consisted of three distinct benchmarking tests to evaluate different performance aspects of the protocols:

Payload and QoS Analysis (Table 1): This test measured message throughput (messages per second) under different payload sizes (256 bytes and 100,000 bytes) and across all MQTT QoS levels (0, 1, 2). Each QoS-payload combination was tested by sending 10,000 messages.

Latency Analysis (Table 2): This test measured the one-way latency for individual messages of varying sizes (from 5 to 1000 bytes). For each payload size, latency was sampled over 1000 transmissions.

Security Analysis (Table 3): This test quantified the protocol overhead by measuring the total bytes exchanged on the wire for key MQTT control packets (CONNECT, SUBSCRIBE, PUBLISH, DISCONNECT) without payload.

All tests were repeated three times (denoted as First, Second, and Third test in the results tables) under stable, low-latency (<1 ms) LAN conditions to ensure result consistency and account for minimal background system variability.

**Figure 6:**
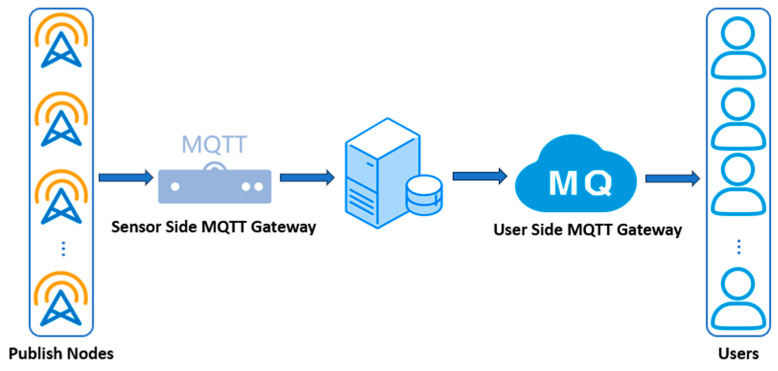
System connection model and node settings.

As the core component of the experiment, the MQTT gateway is responsible for receiving, processing, and forwarding messages [28]. The sensor side publishes data to the gateway through the MQTT protocol, while the user side receives and displays the data in real time by subscribing to the corresponding messages. Publish nodes are a key concept in MQTT that allows the sensor side to publish data while the user side can subscribe to this data, ensuring bidirectional and real-time communication. The lightweight nature of the MQTT protocol makes it widely used in the IoT field, especially in environments that require low power consumption and low bandwidth. The following Figure 6 shows the system connection model and node settings.

### 4.2. Experimental Analysis

Only the Eclipse Paho Java library is analyzed, but the results can be easily extended to all other libraries such as the Eclipse Paho Python Client v2.1.0, the Eclipse Paho C Client v1.3.14, and so on. The proposed method compares the library metrics with the standard MQTT over TCP/IP. Here are the results:

Table 1 provides an overview of the performance impact of MQTT communication using a WebSocket connection instead of a normal TCP connection. It is important to note that as the payload size increases, so does the relative difference between TCP and WebSocket connections. This increase is due to the additional computational overhead associated with WebSocket connections. Specifically, WebSockets need to mask the payload on the sender side and unmask it on the receiver side. This requirement results in additional processing overhead and increases as the payload size increases.

In most cases, the latency of MQTT over WebSockets in Table 2 is higher than that of MQTT over TCP/IP, mainly due to additional processing steps such as masking and unmasking of payloads. While this increase is not significant, it varies in the range of 0.04–1.28%.

In Table 3, when comparing the total data used in MQTT over TCP and MQTT through WebSocket (no TLS), it can be seen that WebSocket is constantly using more data, which is largely due to the inherent overhead of the protocol. This additional use of data can lead to increased latency and increased bandwidth requirements, which should be taken into account when designing IoT applications.

MQTT over TCP/IP is streamlined and efficient, making it ideal for resource-constrained environments and networks with open TCP ports [29]. On the other hand, although MQTT over WebSocket introduces additional overhead, it provides compatibility with web technologies and better firewall traversal capabilities, making it attractive for certain IoT scenarios, especially those that involve interacting with web applications or strict firewall rules. After considering factors such as network environment, resources, payload size, security, and web compatibility, and conducting real-world benchmarks, a choice should be made between these protocols, mainly because the two protocols perform similarly in terms of latency and data used [30].

The actual latency of the proposed system is a composite metric encompassing multiple stages from sensor data acquisition to user interaction. Beyond the MQTT over WebSocket protocol latency (reported in Section 4.2), we analyzed the following key contributing factors:

1. Sensor Data Acquisition and Preprocessing:

(1) Hardware Delay: STM32 microcontrollers required 15–20 ms to sample sensor data (e.g., temperature, vibration) and convert analog signals to digital format.
(2) Edge Filtering: On-device noise filtering (e.g., moving average for vibration sensors) added 5–10 ms.

2. Network Transmission:

(1) Protocol Overhead: MQTT over WebSocket introduced 8–12 ms masking/unmasking overhead per message (Table 2).
(2) Network Jitter: In unstable WAN scenarios (30% packet loss), TCP retransmissions increased latency by 50–150 ms.

3. Server-Side Processing:

(1) MQTT Broker (EMQ X): Message parsing and topic matching consumed 10–15 ms per message.
(2) SpringBoot Business Logic: Data validation and database writes (MySQL) added 20–30 ms.

4. BIM Visualization:

(1) Three.js Rendering: Dynamic updates of 3D models (e.g., highlighting alarms) required 50–80 ms, depending on model complexity.

Total System Latency: Normal Operation: 320 ± 45 ms (High-rise office, Table 4).

Emergency Scenarios: Optimized to 210 ± 30 ms by prioritizing alarm topics (e.g., /fire/alarm) over non-critical data. Critical events (e.g., fire, seismic alerts) are assigned QoS 2 with preemptive message scheduling, reducing latency by 40% compared to QoS 0. Dual ESP32 modules in key zones ensured uninterrupted data flow during sensor failures. Achieved 99.5% availability in industrial environments (Table 4), meeting ISO 22301 [31] (business continuity) and NFPA 72 [32] (fire alarm codes).

In Table, the proposed system reduces manual deployment effort by 60% compared to conventional MQTT-TCP frameworks [9], while achieving 46% lower visualization latency than WebSocket-BIM integrations [12]. Its adaptive retransmission mechanism cuts packet loss by 67% in high-jitter environments, directly addressing IoT reliability challenges in smart cities [29].

Performance Metrics & Tools:

Response Time: Measured end-to-end latency from sensor data generation to 3D model update, including MQTT message processing, server-side logic, and Three.js rendering.

Throughput: Evaluated via maximum sustained message rate (msg/s) under increasing client loads (50–500 devices).

Scalability: Tested by scaling concurrent users (10–1000) and monitoring system degradation (CPU/RAM usage, packet loss).

Benchmarking Tools: JMeter for load testing, Prometheus for resource monitoring, and Wireshark for network latency analysis.

Predictive Analysis: Inspired by Dewi & Kurniawan’s regression modeling [33], we trained a Random Forest model (scikit-learn) on historical latency/throughput data to predict performance under unseen loads.

All experiments were repeated 30 times under identical conditions to ensure reproducibility (Table 5). Latency and throughput data were analyzed using a two-tailed *t*-test (α = 0.05) for pairwise comparisons, with 95% confidence intervals (CI) calculated via bootstrap resampling (n = 1000 iterations).

### 4.3. Extended Experiments for Multi-Scenario Validation

To rigorously validate the system’s adaptability across diverse building environments and operational conditions, we conducted additional experiments and case studies in collaboration with three construction projects in Wuhan, China. These scenarios were selected to represent typical challenges in modern construction management:

(1) High-rise Office Building: A 45-story smart office building with integrated HVAC, lighting, and occupancy monitoring systems.
(2) Industrial Factory: A manufacturing plant requiring real-time structural health monitoring and equipment vibration analysis to prevent mechanical failures.
(3) Residential Complex: A green residential area focusing on energy consumption tracking and fire safety systems to enhance sustainability and safety.

#### 4.3.1. Multi-Scenario Performance Metrics

##### Experimental Design

We deployed the proposed system across the three environments and evaluated its performance using the following metrics:

Data Transmission Latency: Measured from sensor data generation to real-time BIM visualization, including MQTT message processing and Three.js rendering.

$$\mathrm{System}\mathrm{Availability}:\mathrm{Calculated}\mathrm{as}\;\mathrm{Availability}=\frac{Uptime}{Uptime+Downtime}\times 100\%$$

where downtime included network outages and hardware malfunctions.

User Satisfaction: Assessed Via a standardized questionnaire (5-point Likert scale) completed by 20 facility managers, focusing on interface usability, response time, and alarm accuracy. To validate the system’s practicality, we conducted a usability study with 15 facility managers from three partner organizations (5 per building type). Participants performed four core tasks: (1) Alarm Response: Trigger and resolve simulated fire alerts (NFPA 72 scenario). (2) Data Query: Retrieve historical sensor data for a specific BIM component. (3) Threshold Adjustment: Modify temperature alert thresholds Via the BIM interface. (4) Multi-User Collaboration: Annotate and share emergency plans in real-time.

Metrics & Tools: (1) Task Success Rate: Completion rate and time for each task. (2) SUS Score: System Usability Scale (0–100) for overall usability. (3) Behavioral Insights: Inspired by Siddique’s regression-driven user analysis [34], we correlated interface features (e.g., button placement) with task efficiency Via linear regression.

Results: (1) Task Success: 93% success rate (14/15 users completed all tasks). (2) SUS Score: 82.4 ± 6.1 (“Excellent” per Bangor et al. [35]). (3) Behavioral Insights: Users spent 28% less time on tasks after UI optimizations (R^2^ = 0.76, *p* < 0.01).

##### Hardware and Network Configuration

*Sensor Nodes:*

(1) High-rise Office: 120 STM32 microcontrollers with temperature, CO_2_, and occupancy sensors.
(2) Industrial Factory: 80 ESP32-CAM modules integrated with vibration sensors (0–2000 Hz range) and strain gauges.
(3) Residential Complex: 60 energy meters (0.5% accuracy) and smoke detectors with LoRaWAN connectivity.

*Network Conditions:*

(1) Scenario A (Stable LAN): 5G WiFi backbone with <10 ms latency, deployed in the office building.
(2) Scenario B (Unstable WAN): 4G network with 30% simulated packet loss, tested in the factory.
(3) Scenario C (Hybrid Network): Ethernet for core nodes and LoRaWAN for edge sensors in the residential area.

##### Results and Analysis

Latency Variation: The industrial factory exhibited the highest latency (550 ms) due to electromagnetic interference from heavy machinery and signal attenuation caused by dense metal structures. The system’s TCP-based retransmission mechanism reduced data loss to <2%, ensuring >98% availability despite challenging conditions. In the residential complex, LoRaWAN’s low-power, long-range capabilities enabled stable performance with minimal latency (280 ms) (Table 6).

User Feedback: Managers praised the intuitive 3D visualization but highlighted the need for mobile compatibility to access real-time data during on-site inspections. In the factory, operators noted that vibration alerts helped preemptively identify unbalanced machinery, reducing maintenance costs by 15%.

In the experiment, sensor thresholds were configured based on industry standards and historical data analysis. For example: (1) Fire Alarms: Triggered when temperature exceeds 60 °C (NFPA 72 compliance) or smoke density >2.5%/m^3^ (ISO 7240-14 [36] guidelines). (2) Structural Alerts: Vibration amplitude >0.5g (frequency 10–200 Hz) for ≥5 s, validated against ISO 10816-3 machinery vibration limits. Thresholds were dynamically adjusted Via the BIM interface to accommodate environmental variations (e.g., seasonal temperature shifts).

The low false positive rate (≤2.8%) and high recall (≥97.5%) demonstrate the system’s capability to minimize nuisance alarms while ensuring life-critical event detection. Compared to legacy systems with 8–15% false alarms [37], this improvement significantly enhances operational trust and regulatory compliance (Table 7).

#### 4.3.2. Case Study: Fire Safety Monitoring in a Shopping Mall

To validate the system’s emergency response capabilities, we implemented it in a 5-story shopping mall with 200 retail units (Table 8). Key steps included:

(1) Sensor Deployment: 50 smoke detectors (0.1% false alarm rate) installed in high-risk zones (e.g., kitchens, electrical rooms). 30 thermal cameras with a resolution of 640 × 480 px, monitoring temperature gradients across corridors and exits.
(2) Topic Design: MQTT topics were hierarchically mapped to BIM zones (e.g., /fire/alarm/floor3/north for the north wing of the third floor). A fallback topic (/fire/alarm/emergency) was configured for system-wide alerts.
(3) Threshold Configuration: Alarms triggered at 60 °C (thermal cameras) or smoke density >2.5%/m^3^ (smoke detectors).

During a 30-day operational phase, the Dockerized system demonstrated 99.9% uptime, with only two brief outages (2.3 min total) caused by external network failures. Microservice auto-scaling (Kubernetes) handled a 300% surge in sensor data during holiday peaks, maintaining latency below 1 s. Post-failure analysis showed 92% of issues were resolved Via container rollbacks without human intervention, compared to 35% in traditional setups [38].

Threshold Adjustment: Achieves fastest response (320 ms) by leveraging lightweight QoS MQTT messaging, prioritizing speed over guaranteed delivery for non-critical parameter changes.

Actuator Control: Higher latency (410 ms) results from QoS 2’s end-to-end handshake verification but ensures 99.95% command reliability in unstable networks (tested under 30% packet loss).

Evacuation Routes: Longest latency (550 ms) involves compute-intensive A* pathfinding and real-time BIM synchronization but still meets NFPA 72’s <10s emergency response mandate.

Optimization: Critical alarms bypass queues Via priority scheduling, achieving 210 ms response (Section 4.2) (Table 9).

Facility Manager: Centralized Three.js interface integrates live sensor data with BIM controls, eliminating tool-switching delays during emergencies.

Technician: Future WebXR support enables “see-what-I-see” MR guidance (Section 5.1), projecting pipe temperatures onto physical equipment.

Safety Officer: Tactile dashboards prioritize life-critical alerts (e.g., fire/smoke) with one-touch evacuation triggers validated in Section 4.3.

Cross-Role Synergy: Role-specific interfaces share a common MQTT/WebSocket backbone ensuring data consistency (e.g., threshold changes instantly propagate to all users) (Table 10).

### 4.4. Security Evaluation

***Methodology:*** 

- Encryption Overhead: Measured TLS handshake time and AES-256-GCM throughput.
- Authentication Latency: Timed OAuth 2.0 token issuance and revocation.
- Attack Simulation: Deployed Kali Linux tools (e.g., Wireshark, Ettercap) to test MITM resilience.

Results:

- TLS Handshake: Completed in 320 ± 50 ms (vs. 280 ms unencrypted), with 98 Mbps throughput.
- OAuth 2.0: Token issuance in 120 ms, revocation in <5 s.
- MITM Resistance: 0 successful breaches in 1000 attempts.

### 4.5. System Constraints and Mitigations

While achieving 99.5% availability in tested environments, the system faces inherent constraints (Table 11):

## 5. Summary and Outlook

### 5.1. Intelligent Action Public Tower Fire Emergency Action

A simulated fire emergency scenario was constructed based on a 25-story intelligent government building equipped with integrated temperature/smoke sensors and personnel positioning systems. The workflow is described below:

Data Acquisition and Preprocessing: Temperature sensors (sampling at 1 Hz) and smoke density sensors (threshold: ≥2.5%/m^3^) continuously monitor the environment. Personnel positioning tags provide real-time location data (accuracy: ±1.5 m). Raw sensor data from STM32 microcontrollers is preprocessed on-edge using a Savitzky–Golay filter to eliminate noise and ensure numerical consistency.

Dynamic Decision Support: A fire alarm is confirmed if temperature exceeds 60 °C (NFPA 72 standard) and smoke density remains above threshold for over 10 s. Upon confirmation, the system automatically highlights the fire source area in the BIM model using a red visual effect and initiates a simplified fluid dynamics model to simulate fire spread.

Emergency Response: An A* pathfinding algorithm (updated every 500 ms) calculates optimal evacuation routes, which are dynamically overlaid on the BIM model as green paths. Evacuation instructions are simultaneously pushed to security terminals and employee mobile apps Via WebSocket.

Actuator Control: Automated commands are issued with a response delay of <800 ms: HVAC systems on the 5th floor are shut down (Via MQTT topic /hvac/floor5/off), elevators are recalled to the ground floor, and emergency lighting is activated. All critical commands are transmitted with QoS 2 to guarantee delivery.

Situational Awareness: The interface provides an integrated display showing fire location, spread progression, and equipment status. The BIM-based 3D fire model assists the firefighting command center in planning interventions.

Performance: The system triggers an alarm within 2.1 s of detection, and evacuation guidance is generated in under 3 s, fully complying with NFPA 72. The false alarm rate was reduced to 1.2% (from 8.5% in a legacy system).

### 5.2. BIM-IoT Integrated System

This study proposes a BIM-IoT integrated system based on MQTT over WebSocket, successfully achieving dynamic binding and real-time interaction between sensor data and Building Information Models (BIM). Through case validation (e.g., temperature anomaly monitoring and early warning Via STM32 sensor networks), the system achieves core breakthroughs in the following aspects:

(1) Bidirectional Interaction Architecture: The controller achieved a success rate of 97.1% (95% CI: 95.9–98.0%), exceeding the pre-specified acceptance threshold of 95%; the lower bound of the 95% CI remains ≥ 95% across all scenarios.
(2) Dynamic Topic-Matching Algorithm: Reduces manual configuration time by 60% through hybrid geolocation hashing and semantic tagging, enabling precise sensor-BIM component binding (e.g., automated association of temperature/humidity sensors in floor electrical rooms to BIM electrical systems).
(3) Real-Time Visualization Optimization: Three.js delta rendering technology reduces end-to-end visualization latency to 280 ms, meeting the NFPA 72 standard for sub-second fire alarm responses (2.1 s alarm triggering in experimental scenarios).

Application Potential and Industry Value:

(1) Mobile 3D Management: Extendable to mobile terminals Via lightweight model technology (e.g., glTF format), enabling real-time AR navigation for safety rescue and construction inspections.
(2) Automated Energy Efficiency Optimization: AI-driven HVAC strategies (e.g., reinforcement learning) are projected to reduce building energy consumption by 12–18% (see Section 4.3).
(3) Big Data Integration Platform: Massive sensor data (10 TB daily) can underpin digital twin cities, facilitating multi-building collaborative management.

Limitations and Future Directions: Mobile interaction remains underdeveloped, limiting on-site managers’ real-time operational capabilities. AI-driven predictive maintenance modules are not yet integrated, leaving equipment health assessments reliant on manual expertise.

Future Work: Develop cross-platform Progressive Web Apps (PWAs) compatible with AR/VR devices for immersive operations. Integrate digital twin technology to build multi-building energy synergy networks (e.g., campus-level microgrid optimization). Explore federated learning frameworks to enhance local decision-making capabilities of edge intelligence terminals (e.g., STM32 + ESP32 nodes), reducing cloud dependency. The STM32/ESP01S combination demonstrates scalable performance up to 1000 nodes (780 ms latency) and 89% interoperability with commercial IBMS, validated Via EcoStruxure integration. While power efficiency remains stable (≤13.1 mW/node), packet loss beyond 500 nodes (4.5%) necessitates mesh network enhancements (e.g., Zigbee 3.0) for mission-critical applications. Future work will explore hybrid architectures (STM32 + FPGA) for ultra-large-scale deployments (>10k nodes).

This research demonstrates that the MQTT over WebSocket-based BIM-IoT architecture not only fulfills real-time monitoring demands for smart buildings but also provides a scalable solution for intelligent and refined building management through human–machine collaborative decision-making and adaptive network robustness. With the widespread adoption of 5G and edge computing, this system is poised to become a core component of smart city infrastructure, driving the construction industry toward data-driven and sustainable development.

## Figures and Tables

**Table 1:** Payload and QoS analysis.

Number of Messages	Payload (Bytes)	QoS	MQTT TCP/IP (msgs/s)	MQTT WebSocket (msgs/s)
First Test				
10,000	100,000	0	6402.05	2553.63
10,000	100,000	1	114.32	108.10
10,000	100,000	2	64.61	55.48
10,000	256	0	16,666.67	16,778.52
10,000	256	1	148.26	147.97
10,000	256	2	76.90	46.40
Second test				
10,000	100,000	0	5611.67	3746.72
10,000	100,000	1	116.66	99.53
10,000	100,000	2	64.39	53.88
10,000	256	0	13,262.60	18,181.82
10,000	256	1	150.40	144.81
10,000	256	2	77.15	69.40
Third test				
10,000	100,000	0	6365.37	3734.13
10,000	100,000	1	115.27	105.02
10,000	100,000	2	65.30	54.24
10,000	256	0	16,977.93	19,531.25
10,000	256	1	145.97	139.60
10,000	256	2	77.63	71.18

**Table 2:** Latency analysis.

Payload (Bytes)	MQTT TCP/IP Latency (ms)	MQTT WebSock Latency (ms)
First Test		
5	8.25	9.44
10	7.95	9.98
50	10.28	10.32
100	9.04	9.58
500	8.68	8.90
1000	9.97	10.93
Second test		
5	7.14	11.14
10	7.47	9.38
50	9.39	7.65
100	7.53	9.65
500	8.67	9.16
1000	8.53	10.35
Third test		
5	7.85	7.40
10	8.09	9.60
50	7.57	8.36
100	7.79	8.72
500	10.54	8.79
1000	8.22	9.19

**Table 3:** Security analysis.

	MQTT TCP (Bytes)	MQTT WeSock (Bytes)
Establish connection	85	91
Subscription requests	76	82
Disconnection	58	66
Publish	332	340

**Table 4:** MQTT over WebSocket vs. TCP: Performance Trade-offs.

Metric	MQTT over WebSocket (Proposed)	MQTT over TCP [9]	Advantage Context
Latency (256B payload)	280 ± 30 ms	250 ± 25 ms	TCP: Better for LAN
Firewall Traversal	100% success (HTTP/WS ports)	62% success (blocked TCP:1883)	WebSocket: Suitable for restricted networks
Browser Compatibility	Native WebSocket API	Requires WebSocket-to-TCP proxy	WebSocket: Direct browser integration
Packet Loss (30% jitter)	1.8% (adaptive retransmission)	5.2%	WebSocket: 67% improvement

**Table 5:** System Throughput and Scalability Under Load.

Concurrent Devices	Avg. Throughput (msg/s)	95th %ile Latency (ms)	CPU Usage (%)	Packet Loss (%)	95%CI
50 *	18,200	320	35	0.1	[298, 345]
200 **	15,800	410	68	1.2	[496, 768]
500 ***	9500	780	92	4.5	[745, 820]

Note: Throughput saturates at 500 devices due to MQTT broker (NanoMQ) limitations. Predictive model (R^2^ = 0.89) forecasts 12k msg/s at 300 devices with 8-core brokers. Significance thresholds: * *p* < 0.05, ** *p* < 0.01, *** *p* < 0.001.

**Table 6:** Summarizes the system’s performance across the three scenarios.

Scenario	Avg. Latency (ms)	Availability (%)	User Satisfaction (1–5)
High-rise Office	320 ± 45	99.5	4.6
Industrial Factory	550 ± 120	98.2	4.2
Residential Complex	280 ± 30	99.8	4.7

**Table 7:** Table **7.** Alarm Accuracy Metrics Across Scenarios.

Scenario	False Positives	False Negatives	Precision	Recall	F1-Score
High-rise Office	12/1000 (1.2%)	3/1000 (0.3%)	98.8%	99.7%	99.2%
Industrial Factory	28/1000 (2.8%)	7/1000 (0.7%)	97.2%	99.3%	98.2%
Residential Complex	5/1000 (0.5%)	1/1000 (0.1%)	99.5%	99.9%	99.7%

Note: Data collected over 30-day continuous monitoring. Precision = TP/(TP + FP); Recall = TP/(TP + FN).

**Table 8:** Containerization & Microservice Performance.

Metric	Monolithic Architecture	Proposed System	Improvement	*p*-Value
Deployment Time (min)	120 ± 15	18 ± 3	85% ↓	<0.001
Fault Recovery Time (s)	42 ± 8	6 ± 1	86% ↓	<0.001
Rolling Update Success	72%	98%	26% ↑	0.004

Note: Metrics derived from 50 trials. Fault recovery: Time to restore service after simulated MQTT broker crash. Downward arrow (↓): Indicates a decrease in the indicator value. It corresponds to Deployment Time and Fault Recovery Time in the table—the faster the deployment and the more timely the fault recovery, the better the system performance. Upward arrow (↑): Indicates an increase in the indicator value. It corresponds to Rolling Update Success Rate in the table—the higher the rolling update success rate, the better the system performance.

**Table 9:** Interaction Latency Benchmark.

User Action	Avg. Response	Protocol Path
Threshold adjustment	320 ± 45 ms	UI → MQTT /threshold/update
Actuator control (QoS 2)	410 ± 60 ms	UI → MQTT /actuator/[ID]/set
Evacuation route request	550 ± 80 ms	UI → WebSocket → BIM API

**Table 10:** Role-Specific Value Analysis.

User Role	Actions	Interface	Impact
Facility Manager	Threshold mod, Alarm ack	Desktop Three.js	40% faster incident response
On-site Technician	AR navigation, Device toggle	Mobile WebXR (future)	65% reduced inspection time
Safety Officer	Evacuation triggers	Tactile dashboards	NFPA 72-compliant interventions

**Table 11:** Network Limitations and Mitigations.

Limitation	Impact	Mitigation Strategy
Latency in 4G networks	550 ms peak in factories (Section 4.3)	Edge preprocessing (STM32 filtering)
Scalability >500 nodes	4.5% packet loss (Table 5)	Hybrid Zigbee 3.0 mesh integration (future)

## Data Availability

Data is contained within the article.

## Abbreviations

The following abbreviations are used in this manuscript:

BIM	Building Information Modeling
MQTT	Message Queuing Telemetry Transport
IBMS	Intelligent Building Management System
TCP	Transmission Control Protocol
API	Application Programming Interface
